# 5-Cyclo­hexyl-3-(3-fluoro­phenyl­sulfin­yl)-2-methyl-1-benzo­furan

**DOI:** 10.1107/S160053681301475X

**Published:** 2013-06-08

**Authors:** Hong Dae Choi, Pil Ja Seo, Uk Lee

**Affiliations:** a Department of Chemistry, Dongeui University, San 24 Kaya-dong, Busanjin-gu, Busan 614-714, Republic of Korea; b Department of Chemistry, Pukyong National University, 599-1 Daeyeon 3-dong, Nam-gu, Busan 608-737, Republic of Korea

## Abstract

In the title compound, C_21_H_21_FO_2_S, the cyclo­hexyl ring adopts a chair conformation. The 3-fluoro­phenyl ring makes a dihedral angle of 83.16 (4)° with the mean plane [r.m.s. deviation = 0.005 (1) Å] of the benzo­furan ring system. In the crystal, mol­ecules are linked by pairs of C—H⋯π inter­actions into inversion dimers, which are further packed into stacks along the *a-*axis direction by C—H⋯π inter­actions.

## Related literature

For background information and the crystal structures of related compounds, see: Choi *et al.* (2011 ▶, 2012*a* ▶,*b* ▶).
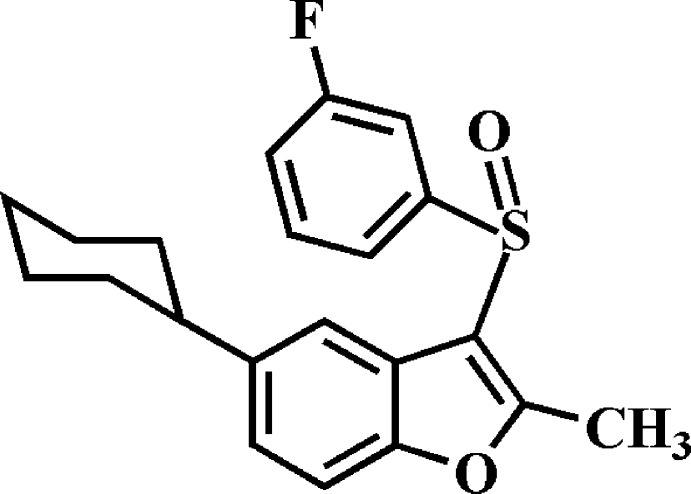


## Experimental

### 

#### Crystal data

C_21_H_21_FO_2_S
*M* *_r_* = 356.44
Triclinic, 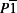

*a* = 8.9147 (1) Å
*b* = 10.1270 (2) Å
*c* = 10.5101 (2) Å
α = 90.376 (1)°
β = 110.407 (1)°
γ = 97.439 (1)°
*V* = 880.44 (3) Å^3^
*Z* = 2
Mo *K*α radiation
μ = 0.21 mm^−1^
*T* = 173 K
0.33 × 0.31 × 0.29 mm

#### Data collection

Bruker SMART APEXII CCD diffractometer
Absorption correction: multi-scan (*SADABS*; Bruker, 2009 ▶) *T*_min_ = 0.692, *T*_max_ = 0.746
19435 measured reflections
4369 independent reflections
3897 reflections with *I* > 2σ(*I*)
*R*_int_ = 0.025

#### Refinement

*R*[*F* ^2^ > 2σ(*F* ^2^)] = 0.042
*wR*(*F* ^2^) = 0.124
*S* = 1.04
4369 reflections
227 parameters
H-atom parameters constrained
Δρ_max_ = 0.94 e Å^−3^
Δρ_min_ = −0.41 e Å^−3^

### 

Data collection: *APEX2* (Bruker, 2009 ▶); cell refinement: *SAINT* (Bruker, 2009 ▶); data reduction: *SAINT*; program(s) used to solve structure: *SHELXS97* (Sheldrick, 2008 ▶); program(s) used to refine structure: *SHELXL97* (Sheldrick, 2008 ▶); molecular graphics: *ORTEP-3 for Windows* (Farrugia, 2012 ▶) and *DIAMOND* (Brandenburg, 1998 ▶); software used to prepare material for publication: *SHELXL97*.

## Supplementary Material

Crystal structure: contains datablock(s) global, I. DOI: 10.1107/S160053681301475X/bx2442sup1.cif

Structure factors: contains datablock(s) I. DOI: 10.1107/S160053681301475X/bx2442Isup2.hkl

Supplementary material file. DOI: 10.1107/S160053681301475X/bx2442Isup3.cml

Additional supplementary materials:  crystallographic information; 3D view; checkCIF report

## Figures and Tables

**Table 1:** Hydrogen-bond geometry (Å, °) *Cg*1 and *Cg*2 are the centroids of the C1/C2/C7/O1/C8 furan ring and the C2–C7 benzene ring, respectively.

*D*—H⋯*A*	*D*—H	H⋯*A*	*D*⋯*A*	*D*—H⋯*A*
C13—H13*A*⋯*Cg*1^i^	0.99	3.00	3.697 (1)	128
C14—H14*B*⋯*Cg*2^i^	0.99	2.91	3.569 (1)	125
C19—H19⋯*Cg*2^ii^	0.95	2.90	3.677 (1)	140

Symmetry codes: (i) 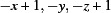; (ii) 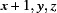.

**Fig. 1.**
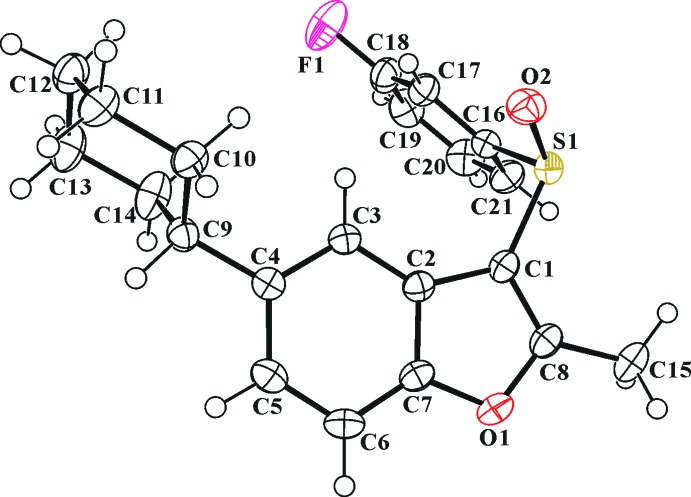
The molecular structure of the title compound with the atom numbering scheme. Displacement ellipsoids are drawn at the 50% probability level. H atoms are presented as small spheres of arbitrary radius.

**Fig. 2.**
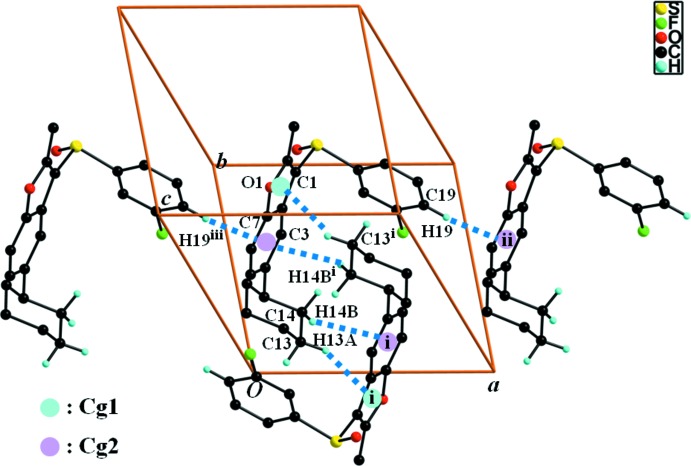
A view of the C—H···π interactions (dotted lines) in the crystal structure of the title compound. H atoms non-participating in hydrogen-bonding were omitted for clarity. [Symmetry codes: (i) - x + 1, - y, - z + 1; (ii) x + 1, y, z; (iii) x - 1, y, z.]

## supplementary crystallographic information

### Comment

As a part of our continuing study of 5-cyclohexyl-2-methyl-1-benzofuran derivatives containing phenylsulfinyl (Choi *et al.*, 2011), 4-bromophenylsulfinyl (Choi *et al.*, 2012*a*) and 4-methylphenylsulfinyl (Choi *et al.*, 2012*b*)substituents in 3-position, we report herein the crystal structure of the title compound.

In the title molecule (Fig. 1), the benzofuran unit is essentially planar, with a mean deviation of 0.005 (1) Å from the least-squares plane defined by the nine constituent atoms. The cyclohexyl ring has the chair form. The dihedral angle between the 3-fluorophenyl ring and the mean plane of the benzofuran ring system is 83.16 (4)°. In the crystal structure (Fig. 2), molecules are connected by pairs of C—H···π interactions into dimers, which are further packed into stacks along the a axis by C—H···π interactions (Table 1, Cg1 and Cg2 are the centroids of the C1/C2/C7/O1/C8 furan ring and the C2–C7 benzene ring, respectively).

### Experimental

3-Chloroperoxybenzoic acid (77%, 202 mg, 0.9 mmol) was added in small portions to a stirred solution of 5-cyclohexyl-3-(3-fluorophenylsulfanyl)-2-methyl-1-benzofuran (272 mg, 0.9 mmol) in dichloromethane (30 mL) at 273 K. After being stirred at room temperature for 4h, the mixture was washed with saturated sodium bicarbonate solution and the organic layer was separated, dried over magnesium sulfate, filtered and concentrated at reduced pressure. The residue was purified by column chromatography (hexane-ethyl acetate, 4:1 v/v) to afford the title compound as a colorless solid [yield 68%, m.p. 403–404 K; *R*_f_ = 0.43 (hexane-ethyl acetate, 4:1 v/v)]. Single crystals suitable for X-ray diffraction were prepared by slow evaporation of a solution of the title compound in ethyl acetate at room temperature.

### Refinement

All H atoms were positioned geometrically and refined using a riding model, with C—H = 0.95 Å for aryl, 1.00 Å for methine, 0.99 Å for methylene and 0.98 Å for methyl H atoms, respectively. *U*_iso_(H) = 1.2*U*_eq_(C) for aryl, methine and methylene, and 1.5*U*_eq_(C) for methyl H atoms. The positions of methyl hydrogens were optimized rotationally.

### Crystal data

**Table:**

C_21_H_21_FO_2_S	*Z* = 2
*M**_r_* = 356.44	*F*(000) = 376
Triclinic, *P*1	*D*_x_ = 1.345 Mg m^−^^3^
Hall symbol: -P 1	Melting point = 403–404 K
*a* = 8.9147 (1) Å	Mo *K*α radiation, λ = 0.71073 Å
*b* = 10.1270 (2) Å	Cell parameters from 9475 reflections
*c* = 10.5101 (2) Å	θ = 2.6–28.3°
α = 90.376 (1)°	µ = 0.21 mm^−^^1^
β = 110.407 (1)°	*T* = 173 K
γ = 97.439 (1)°	Block, colourless
*V* = 880.44 (3) Å^3^	0.33 × 0.31 × 0.29 mm

### Data collection

**Table:**

Bruker SMART APEXII CCD diffractometer	4369 independent reflections
Radiation source: rotating anode	3897 reflections with *I* > 2σ(*I*)
Graphite multilayer monochromator	*R*_int_ = 0.025
Detector resolution: 10.0 pixels mm^-1^	θ_max_ = 28.3°, θ_min_ = 2.0°
φ and ω scans	*h* = −11→11
Absorption correction: multi-scan (*SADABS*; Bruker, 2009)	*k* = −13→13
*T*_min_ = 0.692, *T*_max_ = 0.746	*l* = −13→14
19435 measured reflections	

### Refinement

**Table:**

Refinement on *F*^2^	Primary atom site location: structure-invariant direct methods
Least-squares matrix: full	Secondary atom site location: difference Fourier map
*R*[*F*^2^ > 2σ(*F*^2^)] = 0.042	Hydrogen site location: difference Fourier map
*wR*(*F*^2^) = 0.124	H-atom parameters constrained
*S* = 1.04	*w* = 1/[σ^2^(*F*_o_^2^) + (0.0748*P*)^2^ + 0.2924*P*] where *P* = (*F*_o_^2^ + 2*F*_c_^2^)/3
4369 reflections	(Δ/σ)_max_ < 0.001
227 parameters	Δρ_max_ = 0.94 e Å^−^^3^
0 restraints	Δρ_min_ = −0.41 e Å^−^^3^

### Special details

**Table:**

Geometry. All esds (except the esd in the dihedral angle between two l.s. planes) are estimated using the full covariance matrix. The cell esds are taken into account individually in the estimation of esds in distances, angles and torsion angles; correlations between esds in cell parameters are only used when they are defined by crystal symmetry. An approximate (isotropic) treatment of cell esds is used for estimating esds involving l.s. planes.
Refinement. Refinement of F^2^ against ALL reflections. The weighted R-factor wR and goodness of fit S are based on F^2^, conventional R-factors R are based on F, with F set to zero for negative F^2^. The threshold expression of F^2^ > 2sigma(F^2^) is used only for calculating R-factors(gt) etc. and is not relevant to the choice of reflections for refinement. R-factors based on F^2^ are statistically about twice as large as those based on F, and R- factors based on ALL data will be even larger.

### Fractional atomic coordinates and isotropic or equivalent isotropic displacement parameters (Å^2^)

**Table:**

	*x*	*y*	*z*	*U*_iso_*/*U*_eq_	
S1	0.65884 (4)	0.48559 (3)	0.79845 (3)	0.02628 (11)	
F1	0.84222 (15)	0.35909 (14)	0.41074 (11)	0.0615 (4)	
O1	0.47089 (13)	0.17436 (11)	0.94217 (10)	0.0312 (2)	
O2	0.54304 (14)	0.54929 (11)	0.68662 (11)	0.0358 (3)	
C1	0.55705 (16)	0.33517 (14)	0.82691 (13)	0.0252 (3)	
C2	0.44498 (15)	0.23532 (13)	0.72755 (13)	0.0235 (3)	
C3	0.38240 (16)	0.21856 (13)	0.58605 (13)	0.0242 (3)	
H3	0.4136	0.2834	0.5317	0.029*	
C4	0.27338 (16)	0.10501 (13)	0.52593 (13)	0.0248 (3)	
C5	0.22910 (18)	0.01022 (15)	0.60832 (15)	0.0307 (3)	
H5	0.1549	−0.0669	0.5659	0.037*	
C6	0.28993 (18)	0.02523 (16)	0.74949 (15)	0.0323 (3)	
H6	0.2597	−0.0393	0.8045	0.039*	
C7	0.39658 (16)	0.13922 (14)	0.80477 (13)	0.0268 (3)	
C8	0.56642 (17)	0.29422 (15)	0.95219 (14)	0.0281 (3)	
C9	0.20412 (16)	0.08110 (14)	0.37274 (14)	0.0264 (3)	
H9	0.1189	0.0013	0.3508	0.032*	
C10	0.12469 (18)	0.19886 (15)	0.30242 (14)	0.0292 (3)	
H10A	0.0361	0.2136	0.3349	0.035*	
H10B	0.2054	0.2804	0.3272	0.035*	
C11	0.05690 (19)	0.17497 (17)	0.14790 (15)	0.0341 (3)	
H11A	−0.0336	0.1006	0.1225	0.041*	
H11B	0.0138	0.2558	0.1061	0.041*	
C12	0.18461 (19)	0.14180 (17)	0.09251 (15)	0.0350 (3)	
H12A	0.1342	0.1200	−0.0066	0.042*	
H12B	0.2683	0.2205	0.1072	0.042*	
C13	0.2628 (2)	0.02487 (19)	0.16166 (16)	0.0408 (4)	
H13A	0.3497	0.0087	0.1277	0.049*	
H13B	0.1812	−0.0561	0.1386	0.049*	
C14	0.3336 (2)	0.05058 (19)	0.31578 (16)	0.0396 (4)	
H14A	0.4218	0.1269	0.3393	0.048*	
H14B	0.3803	−0.0288	0.3581	0.048*	
C15	0.6580 (2)	0.35137 (18)	1.09261 (15)	0.0377 (4)	
H15A	0.6953	0.4463	1.0897	0.057*	
H15B	0.7515	0.3045	1.1339	0.057*	
H15C	0.5878	0.3410	1.1468	0.057*	
C16	0.79260 (16)	0.41782 (13)	0.72905 (13)	0.0248 (3)	
C17	0.75975 (17)	0.41440 (15)	0.59050 (14)	0.0291 (3)	
H17	0.6656	0.4446	0.5295	0.035*	
C18	0.8701 (2)	0.36513 (17)	0.54482 (16)	0.0354 (3)	
C19	1.0094 (2)	0.32320 (17)	0.62948 (18)	0.0398 (4)	
H19	1.0829	0.2907	0.5939	0.048*	
C20	1.0398 (2)	0.32951 (18)	0.76767 (18)	0.0404 (4)	
H20	1.1352	0.3006	0.8281	0.049*	
C21	0.93245 (18)	0.37764 (17)	0.81917 (15)	0.0344 (3)	
H21	0.9542	0.3830	0.9143	0.041*	

### Atomic displacement parameters (Å^2^)

**Table:**

	*U*^11^	*U*^22^	*U*^33^	*U*^12^	*U*^13^	*U*^23^
S1	0.02997 (19)	0.02451 (18)	0.02462 (17)	0.00348 (13)	0.01014 (13)	−0.00131 (12)
F1	0.0640 (7)	0.0929 (10)	0.0356 (5)	0.0216 (7)	0.0239 (5)	−0.0039 (6)
O1	0.0346 (5)	0.0388 (6)	0.0242 (5)	0.0057 (4)	0.0152 (4)	0.0056 (4)
O2	0.0386 (6)	0.0332 (6)	0.0378 (6)	0.0126 (5)	0.0136 (5)	0.0070 (4)
C1	0.0261 (6)	0.0285 (6)	0.0226 (6)	0.0049 (5)	0.0103 (5)	0.0001 (5)
C2	0.0227 (6)	0.0254 (6)	0.0248 (6)	0.0054 (5)	0.0109 (5)	0.0021 (5)
C3	0.0245 (6)	0.0247 (6)	0.0244 (6)	0.0038 (5)	0.0097 (5)	0.0029 (5)
C4	0.0230 (6)	0.0247 (6)	0.0271 (6)	0.0042 (5)	0.0091 (5)	0.0019 (5)
C5	0.0284 (7)	0.0274 (7)	0.0357 (7)	−0.0009 (5)	0.0125 (6)	0.0020 (6)
C6	0.0331 (7)	0.0321 (7)	0.0355 (7)	0.0014 (6)	0.0177 (6)	0.0089 (6)
C7	0.0269 (6)	0.0319 (7)	0.0252 (6)	0.0058 (5)	0.0132 (5)	0.0042 (5)
C8	0.0287 (6)	0.0343 (7)	0.0245 (6)	0.0079 (6)	0.0121 (5)	0.0012 (5)
C9	0.0254 (6)	0.0253 (6)	0.0261 (6)	0.0021 (5)	0.0067 (5)	−0.0004 (5)
C10	0.0319 (7)	0.0306 (7)	0.0275 (6)	0.0093 (6)	0.0118 (5)	0.0020 (5)
C11	0.0338 (7)	0.0420 (8)	0.0276 (7)	0.0119 (6)	0.0099 (6)	0.0039 (6)
C12	0.0371 (8)	0.0417 (8)	0.0284 (7)	0.0035 (6)	0.0149 (6)	−0.0027 (6)
C13	0.0418 (8)	0.0500 (10)	0.0311 (7)	0.0180 (7)	0.0095 (6)	−0.0080 (7)
C14	0.0349 (8)	0.0532 (10)	0.0305 (7)	0.0205 (7)	0.0063 (6)	−0.0063 (7)
C15	0.0426 (8)	0.0493 (9)	0.0229 (6)	0.0084 (7)	0.0130 (6)	−0.0009 (6)
C16	0.0260 (6)	0.0229 (6)	0.0255 (6)	0.0021 (5)	0.0096 (5)	0.0016 (5)
C17	0.0283 (7)	0.0322 (7)	0.0252 (6)	0.0040 (5)	0.0076 (5)	0.0013 (5)
C18	0.0383 (8)	0.0393 (8)	0.0308 (7)	0.0021 (6)	0.0162 (6)	−0.0039 (6)
C19	0.0343 (8)	0.0400 (9)	0.0499 (9)	0.0083 (7)	0.0199 (7)	−0.0028 (7)
C20	0.0310 (8)	0.0447 (9)	0.0449 (9)	0.0129 (7)	0.0098 (7)	0.0071 (7)
C21	0.0328 (7)	0.0406 (8)	0.0278 (7)	0.0089 (6)	0.0069 (6)	0.0061 (6)

### Geometric parameters (Å, º)

**Table:**

S1—O2	1.4831 (11)	C11—C12	1.519 (2)
S1—C1	1.7523 (15)	C11—H11A	0.9900
S1—C16	1.7992 (14)	C11—H11B	0.9900
F1—C18	1.3424 (18)	C12—C13	1.512 (2)
O1—C8	1.3703 (18)	C12—H12A	0.9900
O1—C7	1.3842 (16)	C12—H12B	0.9900
C1—C8	1.3609 (19)	C13—C14	1.526 (2)
C1—C2	1.4520 (18)	C13—H13A	0.9900
C2—C7	1.3920 (19)	C13—H13B	0.9900
C2—C3	1.3948 (17)	C14—H14A	0.9900
C3—C4	1.3925 (19)	C14—H14B	0.9900
C3—H3	0.9500	C15—H15A	0.9800
C4—C5	1.406 (2)	C15—H15B	0.9800
C4—C9	1.5149 (18)	C15—H15C	0.9800
C5—C6	1.390 (2)	C16—C17	1.3809 (18)
C5—H5	0.9500	C16—C21	1.3891 (19)
C6—C7	1.377 (2)	C17—C18	1.379 (2)
C6—H6	0.9500	C17—H17	0.9500
C8—C15	1.4852 (19)	C18—C19	1.373 (2)
C9—C10	1.5281 (19)	C19—C20	1.381 (2)
C9—C14	1.536 (2)	C19—H19	0.9500
C9—H9	1.0000	C20—C21	1.387 (2)
C10—C11	1.5277 (19)	C20—H20	0.9500
C10—H10A	0.9900	C21—H21	0.9500
C10—H10B	0.9900		
			
O2—S1—C1	107.99 (7)	C10—C11—H11B	109.2
O2—S1—C16	107.08 (6)	H11A—C11—H11B	107.9
C1—S1—C16	98.41 (6)	C13—C12—C11	110.99 (13)
C8—O1—C7	106.54 (11)	C13—C12—H12A	109.4
C8—C1—C2	107.28 (12)	C11—C12—H12A	109.4
C8—C1—S1	124.17 (11)	C13—C12—H12B	109.4
C2—C1—S1	128.51 (10)	C11—C12—H12B	109.4
C7—C2—C3	119.47 (12)	H12A—C12—H12B	108.0
C7—C2—C1	104.60 (11)	C12—C13—C14	111.35 (13)
C3—C2—C1	135.92 (13)	C12—C13—H13A	109.4
C4—C3—C2	118.74 (12)	C14—C13—H13A	109.4
C4—C3—H3	120.6	C12—C13—H13B	109.4
C2—C3—H3	120.6	C14—C13—H13B	109.4
C3—C4—C5	119.68 (13)	H13A—C13—H13B	108.0
C3—C4—C9	120.75 (12)	C13—C14—C9	111.27 (12)
C5—C4—C9	119.56 (12)	C13—C14—H14A	109.4
C6—C5—C4	122.44 (13)	C9—C14—H14A	109.4
C6—C5—H5	118.8	C13—C14—H14B	109.4
C4—C5—H5	118.8	C9—C14—H14B	109.4
C7—C6—C5	116.01 (13)	H14A—C14—H14B	108.0
C7—C6—H6	122.0	C8—C15—H15A	109.5
C5—C6—H6	122.0	C8—C15—H15B	109.5
C6—C7—O1	125.68 (13)	H15A—C15—H15B	109.5
C6—C7—C2	123.65 (13)	C8—C15—H15C	109.5
O1—C7—C2	110.67 (12)	H15A—C15—H15C	109.5
C1—C8—O1	110.90 (12)	H15B—C15—H15C	109.5
C1—C8—C15	133.45 (15)	C17—C16—C21	121.91 (13)
O1—C8—C15	115.64 (13)	C17—C16—S1	119.95 (11)
C4—C9—C10	111.98 (11)	C21—C16—S1	118.02 (10)
C4—C9—C14	111.49 (11)	C18—C17—C16	116.83 (13)
C10—C9—C14	109.86 (12)	C18—C17—H17	121.6
C4—C9—H9	107.8	C16—C17—H17	121.6
C10—C9—H9	107.8	F1—C18—C19	117.70 (14)
C14—C9—H9	107.8	F1—C18—C17	118.79 (15)
C11—C10—C9	111.76 (12)	C19—C18—C17	123.50 (14)
C11—C10—H10A	109.3	C18—C19—C20	118.24 (14)
C9—C10—H10A	109.3	C18—C19—H19	120.9
C11—C10—H10B	109.3	C20—C19—H19	120.9
C9—C10—H10B	109.3	C19—C20—C21	120.67 (14)
H10A—C10—H10B	107.9	C19—C20—H20	119.7
C12—C11—C10	111.91 (12)	C21—C20—H20	119.7
C12—C11—H11A	109.2	C20—C21—C16	118.81 (14)
C10—C11—H11A	109.2	C20—C21—H21	120.6
C12—C11—H11B	109.2	C16—C21—H21	120.6
			
O2—S1—C1—C8	−134.55 (12)	C7—O1—C8—C15	179.79 (12)
C16—S1—C1—C8	114.34 (12)	C3—C4—C9—C10	−54.46 (17)
O2—S1—C1—C2	42.59 (13)	C5—C4—C9—C10	126.65 (14)
C16—S1—C1—C2	−68.53 (13)	C3—C4—C9—C14	69.09 (17)
C8—C1—C2—C7	−0.61 (14)	C5—C4—C9—C14	−109.80 (15)
S1—C1—C2—C7	−178.13 (10)	C4—C9—C10—C11	179.30 (12)
C8—C1—C2—C3	179.01 (14)	C14—C9—C10—C11	54.84 (16)
S1—C1—C2—C3	1.5 (2)	C9—C10—C11—C12	−54.84 (17)
C7—C2—C3—C4	−0.38 (19)	C10—C11—C12—C13	54.65 (18)
C1—C2—C3—C4	−179.96 (14)	C11—C12—C13—C14	−55.75 (19)
C2—C3—C4—C5	−0.09 (19)	C12—C13—C14—C9	57.1 (2)
C2—C3—C4—C9	−178.99 (11)	C4—C9—C14—C13	179.29 (14)
C3—C4—C5—C6	0.2 (2)	C10—C9—C14—C13	−55.97 (18)
C9—C4—C5—C6	179.11 (13)	O2—S1—C16—C17	−9.39 (14)
C4—C5—C6—C7	0.2 (2)	C1—S1—C16—C17	102.44 (12)
C5—C6—C7—O1	179.75 (13)	O2—S1—C16—C21	166.74 (12)
C5—C6—C7—C2	−0.7 (2)	C1—S1—C16—C21	−81.42 (13)
C8—O1—C7—C6	−179.98 (14)	C21—C16—C17—C18	2.0 (2)
C8—O1—C7—C2	0.40 (15)	S1—C16—C17—C18	177.96 (11)
C3—C2—C7—C6	0.8 (2)	C16—C17—C18—F1	179.49 (14)
C1—C2—C7—C6	−179.50 (13)	C16—C17—C18—C19	−1.5 (2)
C3—C2—C7—O1	−179.57 (11)	F1—C18—C19—C20	179.71 (16)
C1—C2—C7—O1	0.13 (14)	C17—C18—C19—C20	0.7 (3)
C2—C1—C8—O1	0.90 (15)	C18—C19—C20—C21	−0.3 (3)
S1—C1—C8—O1	178.55 (9)	C19—C20—C21—C16	0.8 (3)
C2—C1—C8—C15	−179.85 (15)	C17—C16—C21—C20	−1.7 (2)
S1—C1—C8—C15	−2.2 (2)	S1—C16—C21—C20	−177.77 (13)
C7—O1—C8—C1	−0.81 (15)		

### Hydrogen-bond geometry (Å, º)

Cg1 and Cg2 are the centroids of the C1/C2/C7/O1/C8 furan ring and the C2–C7 benzene ring, respectively.

**Table:**

*D*—H···*A*	*D*—H	H···*A*	*D*···*A*	*D*—H···*A*
C13—H13*A*···*Cg*1^i^	0.99	3.00	3.697 (1)	128
C14—H14*B*···*Cg*2^i^	0.99	2.91	3.569 (1)	125
C19—H19···*Cg*2^ii^	0.95	2.90	3.677 (1)	140

Symmetry codes: (i) −*x*+1, −*y*, −*z*+1; (ii) *x*+1, *y*, *z*.
